# Polysaccharide-Based Nanogels to Overcome Mucus, Skin, Cornea, and Blood-Brain Barriers: A Review

**DOI:** 10.3390/pharmaceutics15102508

**Published:** 2023-10-23

**Authors:** Ju Wang, Marco Viola, Claudia Migliorini, Luca Paoletti, Silvia Arpicco, Chiara Di Meo, Pietro Matricardi

**Affiliations:** 1 Department of Drug Chemistry and Technologies, Sapienza University of Rome, 00185 Roma, Italy; ju.wang@uniroma1.it (J.W.); m.viola@uniroma1.it (M.V.); claudia.migliorini@uniroma1.it (C.M.); luca.paoletti@uniroma1.it (L.P.); chiara.dimeo@uniroma1.it (C.D.M.); 2 Department of Drug Science and Technology, University of Turin, 10125 Turin, Italy; silvia.arpicco@unito.it

**Keywords:** polysaccharides, nanogel, biological barriers, nanocarriers, drug delivery

## Abstract

Nanocarriers have been extensively developed in the biomedical field to enhance the treatment of various diseases. However, to effectively deliver therapeutic agents to desired target tissues and enhance their pharmacological activity, these nanocarriers must overcome biological barriers, such as mucus gel, skin, cornea, and blood-brain barriers. Polysaccharides possess qualities such as excellent biocompatibility, biodegradability, unique biological properties, and good accessibility, making them ideal materials for constructing drug delivery carriers. Nanogels, as a novel drug delivery platform, consist of three-dimensional polymer networks at the nanoscale, offering a promising strategy for encapsulating different pharmaceutical agents, prolonging retention time, and enhancing penetration. These attractive properties offer great potential for the utilization of polysaccharide-based nanogels as drug delivery systems to overcome biological barriers. Hence, this review discusses the properties of various barriers and the associated constraints, followed by summarizing the most recent development of polysaccharide-based nanogels in drug delivery to overcome biological barriers. It is expected to provide inspiration and motivation for better design and development of polysaccharide-based drug delivery systems to enhance bioavailability and efficacy while minimizing side effects.

## 1. Introduction

Over the last couple of decades, the field of drug delivery has gained increasing attention from scientists, industry, and clinical doctors. The successful treatment of a wide range of disease states greatly depends on the direct delivery of drugs to a specific site. However, any pharmaceutical agents transported through different delivery routes will inevitably encounter biological barriers before reaching at the disease site, which may reduce the therapeutic effects of drugs [1]. Biological barriers, such as mucus gel, stratum corneum, cornea, and blood-brain barriers, represent one of the most important protection systems of the human body, with an intrinsic function of protecting the internal organs and tissues from invasion of harmful substances (e.g., bacteria, viruses, chemicals, and dust) [2]. Therefore, it presents substantial challenges for the delivery of therapeutic molecules, especially in topical skin and ocular administration [3].

There are two major obstacles to therapeutic drugs reaching the desirable tissue: poor stability and limited transport across biological barriers [4]. Despite the advancements in pharmaceutical research on drug delivery, various administration routes remain formidable challenges in crossing biological barriers and improving therapeutic effects [5]. For example, the skin and cornea barriers must be overcome when the bioactive molecules are administered topically [6]. Generally, pharmaceutical molecules without specific targeting functions have low bioavailability and subsequent low pharmacological activity. In this respect, several nanocarriers have been extensively developed as novel drug delivery systems (DDSs) to overcome such barriers, such as liposomes, polymeric micelles, and nanogels (NGs) [4]. Among these, NGs have been recognized as a new type of advanced delivery carrier. The high water content in the internal three-dimensional network of NGs supports their structural flexibility, and allows the high encapsulation of therapeutic agents in the internal structure and minimizes the damage of active ingredients by external conditions [7]. Furthermore, this unique property also endows an excellent soft rheological nature, deformability, controllable stability, physicochemical tunability, and stimuli-responsive capacity to external conditions [8,9,10]. The smaller particle size and larger specific surface area of NGs can facilitate the effective crossing of biological barriers and remain stable in the in vivo circulation for an extended time, enabling the drugs to be escorted to the targeted tissues. NG-based carriers have been extensively investigated as smart modalities to cross biological barriers for improving drug delivery performance [11].

Polysaccharides are a class of carbohydrates with complex and large molecular structures formed by condensation of several monosaccharide molecules, obtained from a wide range of natural sources such as plants, animals, and microbes [12], and have become a subject of interest in constructing DDSs due to their outstanding advantages [13]. The natural source of polysaccharides endows them with accessibility, good biocompatibility, and low immunogenicity. In addition, the available reactive functional groups of polysaccharide structures (e.g., hydroxyl, carboxyl, and amine) allow chemical modifications [14,15]. Moreover, polysaccharides with unique functions can provide specific binding to cell receptors, achieve stimulus-responsive degradation or release, and enhance tissue penetration, all of which enable them to improve drug delivery efficiency [16,17].

Recently, with the rapid development of nanotechnology, an increasing number of researchers have aimed to overcome biological barriers that impede effective drug delivery using nanogel-based carriers [11]. As polysaccharides play an important role in nanocarrier development, a comprehensive depiction of the recent approaches to crossing these barriers is desirable. However, to the best of our knowledge, few reviews have focused on strategies for overcoming biological barriers via polysaccharide-based NGs [18,19,20]. These published reviews on polysaccharide-based nanogels mainly focused on their biomedical applications [18], their role in drug delivery [19], or their application in crossing the blood retinal barrier [20]. We expect that this review will provide information about recent advances in overcoming key biological barriers (mucus gel, skin, ocular, and blood-brain barriers) using polysaccharide-based NGs, in addition to providing further perspectives on the opportunities and challenges for further clinical applications of NGs.

## 2. Polysaccharide-Based Nanogels and Recent Advancement

The use of polysaccharides for the development of hydrogels has gained attention due to their exceptional biocompatibility and biodegradability. In comparison to other polymer carriers, polysaccharides offer substantial potential and advantages, including accessibility, cost-efficiency, the generation of harmless degradation by-products, and the facilitation of large-scale production. Polysaccharide-based hydrogels are typically prepared from chitosan, cellulose, starch, alginate, hyaluronic acid, guar, etc., and their derivatives. The drug carriers formulated from these natural polymers are regarded as ideal materials. Physical and chemical methods can be employed to crosslink these natural polymers to form hydrogels, including the utilization of chemical crosslinkers and physical radiation [21]. The self-assembly properties of polymers can also be harnessed to prepare hydrogels via non-covalent interactions, including hydrogen bonding, ionic bonding, π-stacking, van der Waals forces, electrostatic forces, and hydrophobic interactions. Although polysaccharide-based hydrogels have been widely developed for the applications such as wound healing [22], tissue engineering [23], and controlled drug delivery [24], as well as in the food field [25], nanogels offer a solution to the size-related limitations often encountered with conventional hydrogels.

Polysaccharide-based nanogels can be designed to encapsulate various therapeutic substances, such as small-molecule drugs (including hydrophobic and hydrophilic molecules), proteins/peptides, nucleic acids, and vaccines. The strategies for preparing nanogels and loading therapeutics have been summarized in our previous review [26]. For biotherapeutics delivery, the potential of polysaccharide-based nanogels is based on their ability to maintain bioactivity and achieve sustained and/or controlled drug release, resulting in reducing the need for frequent administration [24]. Recently, Abdi et al. reported a design of dynamic nanogels constructed entirely from polysaccharides [27]. These dynamically reconfigurable nanogels rely on the interaction between phenylboronic acid (PBA) and sugar moieties, which self-assemble in physiological conditions and are able to combine the biocompatible nature of polysaccharides with the capacities of their crosslinking chemistry. These dynamic nanogels are stable, pH responsive, and effectively internalized by tumor cells. Polysaccharide chains have abundant modifiable groups (e.g., carboxyl, hydroxy, amino, etc.), which can facilitate the functionalization of polysaccharide-based nanogels with capacities of stimulus response. The design of smart drug delivery carriers is especially attractive for cancer therapy, in which these stimulus-response conditions include internal stimuli (e.g., pH, redox, enzyme, and temperature) and external stimuli (magnetic fields, ultrasound, light, temperature, etc.) [28]. Certain polysaccharides exhibit distinctive attributes, exemplified by the reported antimicrobial activity of chitosan and the unique capability of hyaluronic acid (HA) to specifically target the CD44 receptor, so that overexpression occurs in tumor cells and inflammatory tissue. The drug can be conjugated to HA or encapsulated within HA-based nanogels for intracellular drug delivery via a CD44 receptor-dependent mechanism [29,30]. Polysaccharides and their derivates have been widely developed for cancer therapy using tumor microenvironment-responsive nanogels [18,31,32]. Muraoka et al. reported the use of assembled polysaccharide nanogels as an antigen delivery system prepared from cholesteryl group-modified pullulan (CHP) for clinical cancer immunotherapy [33]. These CHP based nanogels can regulate the function of tumor-associated macrophages, demonstrating a potent inhibitory effect against cancers resistant to other immunotherapies, which is attributed to the improvement in the tumor microenvironment regulated by macrophages.

In clinical practice, intravenous injection is a conventional method of drug administration. However, this approach is accompanied by low bioavailability due to the metabolism of most drugs by the kidneys and liver, and inescapable systemic side effects. To overcome these limitations, polysaccharide-based nanogels have been developed with great interest for topical administration and local therapy [34,35]. For instance, Anahita et al. fabricated pH-sensitive in situ forming injectable nanogels for application in the local treatment of breast cancer. The preparation of nanogels was based on alginate/gelatin hybrid hydrogels containing doxorubicin (DOX)-loaded chitosan/gold nanoparticles (CS/AuNPs) [36]. Topical application of nanogels can help to overcome biological barriers through prolonging the retention time and promoting permeation of drug molecules, resulting in enhancing bioavailability and minimizing side effects. As an emerging nanocarrier, polysaccharide-based nanogels have been investigated to cross the blood retinal barrier [20], blood-brain barrier [37], and stratum corneum barrier [38], and in transvascular drug delivery [39]. Due to their high drug-loading capacity, biological stability, and targeting of specific sites, polysaccharide-based nanogels show promise in overcoming various biological barriers through a variety of strategies, which are summarized in this review.

## 3. Polysaccharide-Based NGs to Overcome the Mucus Gel Barrier

### 3.1. Characteristics of Mucus Gel Barrier

**Figure 1:**
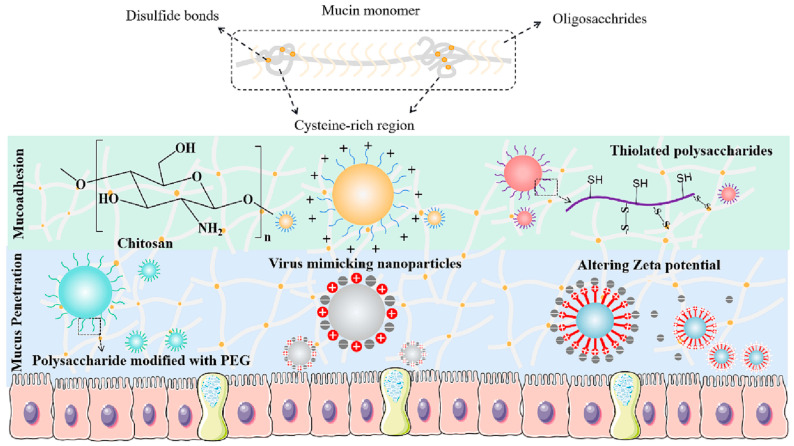
Schematic diagram of muco-adhesion and mucus permeation properties of polysaccharide-based nanoparticles. Reprinted with permission from Yuan et al. [42]. Copyright 2023 Carbohydrate Polymers.

Mucus lines all mucosal surfaces of the body, including the gastrointestinal tract, airways, eyes, cervico-vaginal tract, and the dermis. It forms a potential barrier to mucosal drug delivery. Mucus is a polyelectrolyte gel mainly composed of water (up to 95%) and mucins, which comprise high-molecular-weight glycoproteins forming a fibrous network abundant in proline, threonine, and serine units [40]. The presence of the carboxyl and sulfate groups imparts a negative charge to them. This negative charge, combined with hydrophobic domains, results in mucus being hydrophilic overall. This hydrophilic nature of mucus creates an interactive steric barrier that restricts the free movement of substances within and through the mucus. Moreover, mucus is also a highly complex dynamic gel barrier to drug delivery due to its continuous secretion and shedding from the mucosal surfaces [41]. The size of the pores (around 100 to 200 nm), as well as the composition and structure of the mucus gels, including the covalent and non-covalent interaction forces, play crucial roles in determining how components diffuse through the mucus layer. As illustrated in the schematic diagram (Figure 1), strategies such as muco-adhesion and mucus penetration are efficient approaches to overcoming the mucus barrier using nano formulations.

Given the pore sizes of mucus gel barriers, only nano formulations within a specific size range have the potential to penetrate without modifying the gel layer. Polysaccharide-based nanogels have been widely employed to overcome mucus barriers, thanks to their small size and physicochemical properties, which allow covalent or noncovalent interactions. Consequently, enhancing the muco-adhesion and mucus permeation capabilities of drug delivery systems are the two primary strategies for overcoming the mucus gel barriers.

### 3.2. Strategies to Overcome the Mucus Gel Barrier

#### 3.2.1. Muco-Adhesive

Excellent muco-adhesive ability is one of the most appealing properties of polysaccharides. This is achieved by various covalent and non-covalent interactions between polymer and mucus, such as hydrogen bonding, disulfide bonding, the entanglement of macromolecules, electrostatic interactions, hydrophobic interactions, and other van der Waals interactions [43]. Muco-adhesive polysaccharides prolong the contact of the formulation with the mucosa surface, thereby increasing the chance for drug absorption and release. Most polysaccharides endow considerable polymer chain lengths, along with an abundance of hydrogen-bonded donors and acceptors (e.g., carboxyl, hydroxyl, and amino groups), which significantly bolster their ability to adhere to mucosal surfaces [44].

Chitosan (CS)-based nanogels are of particular interest. They possess a cationic polyelectrolyte nature, which enables strong electrostatic interactions with mucus or negatively charged mucosal surfaces. These nanogels are capable of achieving a longer residence time in the mucus layer, enhancing cellular uptake, facilitating targeted drug release, and ultimately, boosting anticancer activities [45,46,47,48]. Polysaccharides containing carboxyl groups are partially negatively charged at neutral pH. HA and carboxymethylcellulose (CMC) represent examples of anionic polysaccharides; their adhesion properties, however, may be associated with factors such as hydrogen bonds and van der Waals forces [49]. N. Zoratto et al. reported that HA-based nanogels can effectively extend precorneal retention and enhance the bio-adhesive properties of carriers through interactions with receptors on the corneal epithelium [50]. Due to these properties, polysaccharide-based formulations have been employed as potential carriers for pulmonary delivery [51]. To enhance the muco-adhesive strength of polysaccharides, chemical modification methods are commonly employed. Among these methods, thiolation stands out as one of the most attractive, since polymers containing thiol groups enable an increase in the adhesion strength via the thiol-disulfide bond exchange reaction, leading to covalent bonds with mucus glycoproteins [52]. Thiolated CS derivatives such as chitosan-stearic acid conjugate (CSA) [53], N-acetyl-L-cysteine modified carboxymethyl chitosan (N-CMCS) [54], and folate–CsSH/PEGBCOOH nanogels [55] have been synthesized to enhance the muco-adhesive properties of mucosal drug carriers. Similarly, HA was thiolated and subsequently preactivated with 6-mercaptonicotinamide (HA-CYS–MNA) to enhance stability and muco-adhesive properties on vaginal mucosa [56]. Y. Shtenberg et al. reported that maleimide-terminated PEG modified alginate (Alg) revealed a 3.6-fold increase in adhesion force on mucosa surfaces [57].

#### 3.2.2. Mucus Penetration

The penetration of the mucous layer by the nano drug carrier system is crucial for accessing the underlying epithelium, and represents one of the most effective strategies for enhancing bioavailability in the mucosal drug delivery system. The particle size and charge of nano formulations significantly influence their penetration and uptake. Therefore, the surface chemistry of nanogels can be tailored to promote deep penetration into the mucus gel layer [58]. The negative charge of the mucus layer makes it easier for positively charged nanocarriers to adhere, while negatively charged nanocarriers find it easier to pass through the mucus. However, it is worth noting that positively charged carriers tend to exhibit better endocytosis compared to negatively charged carriers [59]. To enhance the permeability of the mucus layer, an effective strategy is to design nanoplatforms with a net neutral charge and small particle size. Surface modification of carriers with the purpose of endowing the nanocarrier surface with a high and equal density of positive and negative charges, thus minimizing the electrostatic interactions with mucus, is conducive to mucus penetration. For instance, cationic chitosan and its derivatives can be combined with anionic polysaccharides such as sodium alginate, dextran sulfate, or pullulan to improve nasal vaccine delivery [60]. Furthermore, the development of nanocarriers with modified surface charges is an effective strategy to enhance both mucus permeation and cellular endocytosis. Tian et al. prepared core-shell nanocarriers consisting of positively charged nanoparticle cores coated with thiolated hyaluronic acid (HA-SH) to improve oral drug delivery [61]. It has been reported that some viruses with both positive and negative charges can diffuse and penetrate through the mucosal layer into cells [62]. The preparation of nanocarriers that mimic viruses is another effective strategy to enhance mucus penetration. In this way, the dense coating with positive and negative charges can play a role in modulating the interaction of carriers with the mucus layer. Therefore, the surface chemical properties of polysaccharides can be finely regulated by the chemical modification to prepare carriers that enhance mucus penetrability [63]. In addition, the introduction of polyethylene glycol (PEG), a hydrophilic polymer known for its excellent biocompatibility, can further enhance mucus permeability. PEGylated chitosan, for instance, has demonstrated improved stability, membrane permeability, and reduced toxicity in vaccine delivery systems [64] and nasal mucosa delivery [65].

Another feasible strategy to enhance the mobility and permeation of nanogels in mucus is to disrupt the mucus structure. This can be achieved using mucolytic agents such as N-acetylcysteine, which can decorate the nanogels to reduce the mucus viscosity by breaking disulfide bonds and reducing the crosslinking of proteins in the mucus layer. This thereby reduces the interactions between the nanogels and mucus, allowing for improved mobility and permeation [66]. Table 1 summarizes the examples discussed in this section.

## 4. Polysaccharide-Based NGs to Overcome the Skin Barrier

### 4.1. Characteristics of Skin Barrier

**Figure 2:**
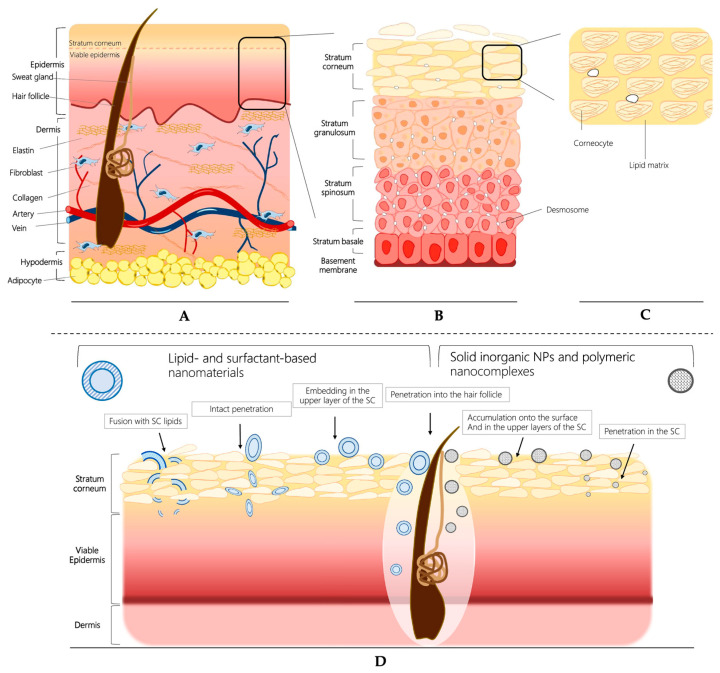
Schematic representation of the (**A**) skin structure, (**B**) epidermis structure, and (**C**) stratum corneum composition (“brick and mortar” structure). (**D**) Potential routes of skin penetration for nanomaterials. Adapted with permission from Salvioni et al. [71]. Copyright 2021 Advances in Colloid and Interface Science.

Topical or transdermal drug delivery is a non-invasive and promising option for the treatment of both skin and certain systemic diseases. This method offers advantages such as bypassing the hepatic first-pass metabolism and avoiding degradation in the gastrointestinal tract. However, effectively delivering therapeutic molecules to the desired site remains a great challenge because of the presence of skin’s natural barrier. Healthy skin naturally provides a highly effective barrier that prevents the entrance of foreign compounds from the external environment. The skin is structured in multiple layers, which can roughly be categorized as the epidermis (comprising the stratum corneum and viable epidermis), dermis, and hypodermis (Figure 2) [67]. The stratum corneum, situated as the outermost layer of the skin, serves as a significant physicochemical and anatomical barrier for both topical and transdermal drug delivery. It is composed of terminally differentiated corneocytes surrounded by extracellular non-polar lipids. As a consequence, this barrier can restrict the penetration of molecules larger than 500 Da [68], creating an immense challenge when attempting to deliver larger active molecules to the skin through passive diffusion. Furthermore, in the situation of inflammatory skin diseases such as psoriasis, atopic dermatitis, and skin cancer the inflamed skin exhibits an impaired skin barrier function and stratum corneum integrity [69]. Inflammatory stimuli can lead to dynamic changes in epidermal tight junctions, such as the hyperproliferation of keratinocytes observed in psoriasis, resulting in thickening of the epidermis [70]. Although the impaired skin barrier may facilitate the penetration of active compounds into lesioned skin, striking the right balance between skin penetration and retention becomes challenging because effective topical drug delivery should not only ensure adequate effectiveness but also minimize systemic side effects.

### 4.2. Strategies to Overcome the Skin Barrier

To enhance the penetration of active compounds into the skin, several traditional strategies have been developed. These include the use of chemical enhancers such as surfactants, alcohols and amines, as well as physical manipulation of the stratum corneum via approaches such as microneedles, electroporation, sonoporation, and iontophoresis [72]. Although these approaches have proven to be effective for the delivery of therapeutics, they may also potentially cause long-term or irreparable damage to the skin’s lipid structure. Polysaccharide-based nanogels, due to certain desirable features, provide an alternative approach to conventional strategies for topical and transdermal drug delivery with minimal damage to the natural barrier function of the skin. Table 2 summarizes the examples discussed in this section.

#### 4.2.1. Enhancement of Permeation and Targeted Delivery

Polysaccharide-based nanogels have gained significant attention in the field of dermal drug delivery due to their favorable properties, including non-toxicity, biodegradability, and biocompatibility. Among these polymers, chitosan, a unique cationic natural polysaccharide, stands out for its flexibility in the design of various formulations for dermal drug delivery due to its remarkable physical and biological properties, including demonstrated antibacterial, antifungal, and antioxidant capabilities [73]. Chitosan and its derivatives have been employed to prepare bio-adhesive gels and nanoparticle-incorporated gels. These formulations serve multiple purposes, including improving drug stability and enhancing drug permeation through the skin barrier [74]. Janna et al. developed aceclofenac-loaded nanoparticles using a combination of chitosan and egg albumin, which were then dispersed in a Carbopol 940-based gel base. The incorporated nanogels showed sustained drug permeation for up to 8 h when tested on the excised mouse skin model. Importantly, both the permeation flux and the anti-inflammatory effect of this formulation were significantly higher compared to those of a commercially available gel [75]. Similarly, a clobetasol-loaded chitin nanogel with the particle size of 132 ± 14 nm showed enhanced transdermal flux and significant anti-psoriasis activity even at a low concentration, which can be attributed to the loosening of epidermal layers [76]. In addition, chitosan-based nanogels prepared using the ionic gelation technique were evaluated by Al-Kassas et al., who demonstrated that these nanogels prolonged drug release and created a drug reservoir after entering the skin [77]. Recently, luliconazole-loaded nanosuspensions prepared from starch ester derivative were incorporated into Carbopol 934 gel. These nanogels exhibited significantly higher skin permeation and increased drug retention in the skin, approximately three times greater than those of standard cream, and hence are particularly useful for treating illnesses caused by resistant fungal strains [78].

Gums, as representatives of polysaccharides used as gel-forming agents, were evaluated in a study. Specifically, xanthan at a concentration of 1% was compared with Carbopol 934 (also at 1%) for incorporating ketoconazole-loaded nanoparticles in the cutaneous delivery of this antifungal drug [79]. The results demonstrated that xanthan exhibited greater bio-adhesive strength compared to Carbopol 934, and a sustained release profile was observed for the nano-based hydrogels. Due to the suitable properties, alginate has been extensively studied in biomedical and pharmaceutical applications. Abnoos et al. investigated sodium alginate and chitosan-based nanogels for transdermal delivery of pirfenidone. Ex vivo permeation studies on mouse skin showed a significant enhancement in drug permeation and sustained release, which could be beneficial for the treatment of idiopathic pulmonary fibrosis [80].

Hyaluronic acid (HA), a major component of the extracellular matrix, has found extensive use in dermatology due to its reported pharmacological and biological properties. Silk peptide–HA-based nanogels have been synthesized for the enhancement of the topical skin delivery of curcumin, effectively improving the skin retention and delivery of drugs to deeper skin layers [74]. Moreover, due to the specific interaction with the CD44 receptor, HA and its derivates are attractive candidates for drug delivery systems targeting inflammatory-related skin diseases where CD44 overexpression occurs, such as psoriasis and atopic dermatitis [81]. Montanari et al. conducted research on HA-cholesterol self-assembled nanogels designed for targeting intracellular *S. aureus* in persistent skin or wound infections. Their work confirmed the CD44-mediated accumulation and cellular uptake of these nanogels [82]. Additionally, Liu et al. developed HA-coated liposome nanogels by combining the advantages of liposomes and HA. In vitro studies of melanoma cells demonstrated that these nanogels could promote the cellular uptake through targeted delivery, leading to the inhibition of tyrosinase activity and melanin production [83]. In another study, Kim et al. constructed HA/β-glucan hybrid nanogels designed for topical dermal delivery and targeting of skin dendritic cells [84]. These nanogels effectively penetrated the stratum corneum and deposited in the dermis via hybridization with HA-methacrylate, and targeted dendritic cells with the assistance of hybridized schizophyllan-methacrylate (SPGMA).

#### 4.2.2. pH-Responsive Nanogel Systems

Environmentally responsive nanogels have emerged as promising carriers for topical and transdermal drug delivery. These nanogels are designed to be sensitive to physical or chemical stimuli, giving them the capability to deliver active compounds in a site-specific manner. In topical applications, pH-responsive nanogels are designed to take advantage of the pH gradient in the natural conditions along the different skin layers. This design facilitates the penetration and subsequent release of cargo molecules into the deeper layers of the skin [85]. In recent decades, various pH-responsive nanogels have been explored to target a range of skin diseases. For instance, Sahu et al. developed a chitosan-based pH-responsive nanogel [86] and a double-walled PLGA-chitosan nanogel [87] encapsulated with 5-Fluorouracil (5-FU) for topical chemotherapy of melanoma. The pH-responsive behavior of these nanogels led to a triggered release of 5-FU in the slightly acidic microenvironment of the tumor site. This selective drug accumulation at the melanoma site was attributed to the properties of cationic nanogels containing amine groups, which protonated at low or acidic pH, thereby enhancing the swelling rate of the nanogels [88]. Similarly, to overcome the limitations of acitretin as a topical formulation, Divya et al. developed a chitin-based nanogel system for loading acitretin and aloe-emodin for topical delivery in psoriasis. These positively charged nanogels exhibited increased swelling and release at acidic pH levels [89].

Furthermore, pH/thermos dual-responsive nanogel systems have been employed for transdermal drug delivery. Examples include chondroitin sulfate-based nanogels for antimicrobial peptide delivery [90], and a biopolymer system composed of chitosan and hyaluronic acid with pH-responsive properties. This system was further improved by incorporating a thermo-responsive poloxamer 407 (PF127) hydrogel for efficient encapsulation of Cortex Moutan [91]. The release behavior of the polysaccharide-based pH-responsive carrier in combination with PF127 was found to depend on the morphology, mechanical properties, and pH stability of the system. In vitro studies showed the highest cumulative drug release (86.5%) over 5 days under mild acidic conditions (pH 6.4).

#### 4.2.3. Thermo-Responsive Nanogel Systems

Temperature-induced drug release is an attractive strategy for drug delivery due to the wide range of applications where temperature variations are naturally present. In normal skin, which exhibits a temperature gradient between 32 and 37 °C, thermo-responsive polymers undergo transitions in response to temperature changes. This leads to the disruption of nanogels and the selective release of active molecules into the stratum corneum or dermis [92]. Responsive polymers with temperature-sensitive blocks in their structures, primarily synthetic polymers such as poly-N-isopropylacrylamide (PNIPAM) [93], oligo ethylene glycol methacrylate (OEGMA) [94], and poly(N-vinylcaprolactam) [95] have been developed for transdermal drug delivery. These polymers can be modified and combined with polysaccharides to create nanogels with stimuli-responsive properties. As an example, in a study by Giulbudagian et al., β-cyclodextrin (βCD) was grafted onto the thermo-responsive polyglycerol (tPG) polymer to enhance topical drug delivery. βCD has a unique affinity toward dexamethasone and can act as a topical penetration enhancer. When combined with the temperature-responsive tPG, this resulted in effective delivery of dexamethasone to the epidermis and dermis of skin. The nanogel carriers increased drug penetration approximately 2.5-fold in the epidermis and around 30-fold in the dermis compared to a commercial cream [96].

Carmona-Moran et al. [97] reported diclofenac-loaded core-shell nanogels with temperature- and pH-responsive properties. These nanogels were prepared using poly(N-vinylcaprolactam)-co-acrylic acid and incorporated with gellan, which is an anionic polysaccharide, and were then used to create solid film formulations. Transwell diffusion experiments showed that the transport of diclofenac increased 6-fold when the temperature was raised from 22 °C to skin surface temperature of 32 °C. This demonstrated the feasibility and effectiveness of this thermo-responsive system for transdermal drug delivery.

Dual- or multi-responsive drug delivery systems are considered the next generation of carriers, characterized by minimal toxicity and improved therapeutic efficacy, which can intelligently release drugs in response to multiple stimuli, such as pH, temperature, or redox conditions [98]. As discussed above, polysaccharide-based dual-responsive (pH/temperature) nanogels have been developed to enhance transdermal drug delivery [90,91].

**Table 2:** Summary of recent studies on polysaccharide-based nanogels for overcoming the skin barrier.

Author	Type of Nanogel	Function and Mechanism	Size/Zeta Potential	Loaded Cargo	Type of Study	Year	Ref.
Chatterjee et al.	Chitosan, hyaluronic acid	Dual-responsive (pH/temperature)	−17 mV	Cortex Moutan	In vitro	2021	[91]
Liu et al.	Hyaluronic acid	Enhance permeation	122 ± 34 nm	Tranexamic acid	In vitro In vivo	2021	[83]
Kim et al.	Hyaluronic acid, β-glucan	Enhance permeation/uptake of dendritic cells	100–300 nm −7 mV	/	In vitro Ex vivo	2021	[84]
Pan et al.	Sugarcane bagasse cellulose	Multi-responsive (pH/temperature/redox)	/	Doxorubicin	In vitro	2021	[98]
Shaikh et al.	Starch polymer, Carbopol	Enhance permeation and skin retention	369.1–745.4 nm	Luliconazole	In vitro Ex vivo	2020	[78]
Montanari et al.	Hyaluronic acid	Enhance permeation, CD44 targeting	/	Antibiotics	In vitro Ex vivo	2020	[82]
Ghaeini-H et al.	Chondroitin sulfate	Dual-responsive (pH/temperature)	181 nm −8.6 mV	Antimicrobial peptide	In vitro	2020	[90]
Sahu et al.	Chitosan	pH response, Enhance permeation	100–180 nm +43.15 mV	5-Fluorouracil	In vitro In vivo	2019	[86,87]
Abnoos et al.	Chitosan-sodium alginate	Enhance permeation	Around 80 nm	Pirfenidone	In vitro Ex vivo	2018	[80]
Giulbudagian et al.	β-cyclodextrin	Thermo-responsive, enhance permeation	/	Dexamethasone	In vitro Ex vivo	2018	[96]
Panonnummal et al.	Chitin	Enhanced permeation	132 ± 14 nm	Clobetasol	In vitro In vivo	2017	[76]
Carmona-Moran et al.	Gellan	Thermo-responsive, enhance permeation	/	Diclofenac	In vitro	2016	[97]
Al-Kassas et al.	Chitosan, poloxamer, Carbopol	Enhance adhesive, permeation, prolonged drug release	less than 500 nm >30 mV	Propranolol	Ex vivo	2016	[77]
Jana et al.	Chitosan, Carbopol 940	Enhance permeation	352.90 nm −22.10 mV	Aceclofenac	Ex vivo In vivo	2014	[75]

## 5. Polysaccharide-Based NGs to Overcome the Ocular Delivery Barrier

### 5.1. Characteristics of the Ocular Barrier

**Figure 3:**
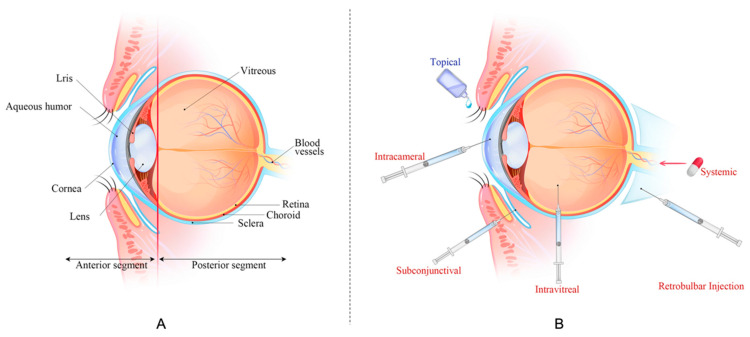
(**A**) The anatomy of the eye, (**B**) routes of drug administration for ocular drug delivery. Reprinted with permission from Li et al. [99]. Copyright 2023 Journal of Nanobiotechnology.

Ocular drug delivery is one of the most challenging issues in ophthalmology duo to the presence of various anatomical and physiological barriers. The eye is a highly complex organ composed of two distinct segments, namely an anterior and a posterior segment (Figure 3) [99]. The anterior segment encompasses the cornea, conjunctiva, lens, iris, and aqueous humor, while the posterior segment contains the retina, choroid, sclera, and vitreous chamber. Anatomically, the ocular tissues are distinct from other tissues in the body, and are protected by unique barriers that can be briefly divided into dynamic and static barriers [100]. Tear turnover, reflex blinking, and nasolacrimal drainage represent the dynamic barriers that prevent foreign substances from reaching the eye surface [100]. The major ocular barriers in the anterior segment include the cornea, tear film, and conjunctiva, which mainly prevent drug delivery to the anterior segment of the eye [101]. The cornea, which has a multilayered structure, acts as the main barrier for foreign substances attempting to enter the anterior chamber. It prevents drug penetration into the parenchyma through its epithelial, stromal, and endothelial layers [99]. The tear film forms a hydrophobic layer on the eye surface, and in combination with the dilution effect of tear fluid, hinders drug absorption when applied topically [102]. Moreover, the blood-retinal barrier (BRB) formed by intercellular tight junctions is the most crucial barrier in the posterior segment of the eye, limiting drug delivery to this area [103].

The conventional routes of ocular drug administration are shown in Figure 3B. Among these, topical administration is the most commonly chosen method due to its advantages, such as non-invasiveness, minimal systemic side effects, and good patient compliance [104]. However, despite advancements in drug delivery and formulation, ocular administration remains a formidable challenge for treating various ocular disorders. Because of the unique physiological and anatomical barriers mentioned above, the bioavailability in ocular drug delivery is usually less than 5% [105]. Therefore, it is essential for novel strategies to overcome these challenges and achieve effective ocular drug delivery.

### 5.2. Strategies for Enhancing Ocular Drug Delivery

In recent years, due to their biocompatibility and high drug encapsulation capacity, polysaccharide-based nanogels have been extensively investigated as ideal carriers for enhancing the bioavailability of drugs in ocular delivery, offering a promising approach for overcoming ocular barriers [20,101]. In topical administration, such as eye drops, it is crucial to maintain a therapeutically relevant drug concentration in the tear film or at the desired site of action. All these strategies involve constructing polysaccharide-based nanogels to either increase the muco-adhesion of the drug on the ocular surface, enhance the corneal and conjunctival permeability of the drug into the eye, or modify nanogels into controlled drug-release systems. Table 3 summarizes the examples discussed in this section.

#### 5.2.1. Enhance the Muco-Adhesion and Permeability

In the context of ocular drug delivery, polysaccharides play a key role in the development of carriers to overcome the barriers due to their excellent physicochemical and biological properties. Many studies on ocular nanocarriers have focused on commercialized polysaccharides, including chitosan, hyaluronic acid, and cellulose, due to their easy accessibility and specific biological functions. Chitosan is a positively charged polysaccharide and can easily bind to the negatively charged surface of the cornea and conjunctiva through electrostatic interaction. This property enhances corneal retention and penetration of the drug [106]. In addition, chitosan has been found to stimulate the migration of keratinocytes, combined with the antibacterial activity, which can contribute to improved corneal wound healing [106]. Buosi et al. prepared chitosan-based nanogels with a ζ-potential value of 32 ± 2 mV by crosslinking with sodium tripolyphosphate for the effective encapsulation of resveratrol [107]. They demonstrated the ability to enhance cellular internalization in human retinal pigment epithelial cells (ARPE-19), resulting in improved antioxidant and anti-inflammatory activities of resveratrol at the specific target site for ocular treatment. Furthermore, polysaccharides are naturally present in the ocular tissue; e.g., hyaluronic acid (HA), a major ingredient of the vitreous humor, has been utilized to mimic the natural lubricating properties of tears and to deliver artificial tear substitutes for the treatment of dry eye syndrome [108].

One of the remarkable properties of HA is its specific binding to the CD44 receptor, which is overexpressed on the epithelial cells of the cornea [109]. This characteristic has led to extensive research into HA and its derivatives for improving ocular drug delivery. For instance, Zoratto et al. developed self-assembling nanogels based on cholesterol-modified hyaluronan, providing a suitable bio-adhesive platform for enhancing drug permeation across the cornea and extending preocular retention time due to their affinity and bio-adhesive properties [50]. Fluorescence-labeled nanogels demonstrated enhanced permeation into the deep layers of the corneal epithelial in porcine corneas, highlighting the strong affinity of these nanogels for ocular tissue. This nanogel system proved effective for delivering hydrophilic drugs such as tobramycin and diclofenac by facilitating their permeation, as well as hydrophobic drugs such as dexamethasone and piroxicam by increasing their preocular retention time. These findings were demonstrated by ex vivo experiments on freshly excised porcine eyeballs [50]. Similarly, Laradji et al. developed redox-responsive nanogels based on hyaluronic acid by the covalent modification of hyaluronic acid to create hyaluronic acid-cystamine-cholesterol conjugate (HA-Cys-CH). These nanogels were designed for topical application with triggered release in a reducing environment, allowing effective delivery of therapeutics to the posterior part of the eye [108]. It is worth noting that the physical characteristics of the drug-loaded system, including small size, negative surface charge, and hydrophilicity, facilitated the ability of nanogels to cross through the negatively charged scleral constituents. This was demonstrated by an increased fluorescence signal observed in the retinal pigmented epithelium (RPE) layer, where a higher expression of CD44 cell receptors occurs in these cells compared to others [108].

It would be a feasible strategy to design hybrid materials containing polysaccharides to maximize the drug delivery capacity of polysaccharide-based nanocarriers. In the nanohybrid system, polysaccharides can improve the biocompatibility and biodegradability of inorganic or synthetic materials and also serve as stabilizers for other compounds such as biological macromolecules [110,111]. Recently, there has been growing interest in macromolecular drugs, gene therapies, and stem cell therapies due to their potential to address complicated ophthalmic diseases through multi-target effects. Chaharband et al. developed chitosan-hyaluronic acid nano-polyplexes loaded with siRNA using the ionic gelation method. Through animal experiments, this nano-system demonstrated its ability to effectively surmount both vitreous and retina barriers, resulting in delivering therapeutic cargo to posterior ocular tissues by intravitreal injection [112]. Silva et al. prepared chitosan- and hyaluronic-acid-based muco-adhesive nanogels for topical ocular delivery of erythropoietin (EPO), utilizing chitosan and hyaluronic acid [113]. The nanogels, which were a combination of CS and HA with a 300 kDa molecular weight, at a 1:1 mass ratio, exhibited superior muco-adhesive properties. In vitro permeation experiments, using fresh porcine corneas, revealed rapid penetration into the conjunctiva, followed by the sclera and cornea, all without causing cytotoxicity [113]. This polysaccharide-based formulation shows promise for enhancing the ocular bioavailability of therapeutics by extending their retention time and facilitating penetration through various ocular membranes.

#### 5.2.2. Stimuli-Responsive in situ Gelling Systems

Overcoming the dynamic barriers of ocular tissues, including the presence of eye fluids, stands as a paramount challenge in augmenting the bioavailability of topically administered medications. One of the most critical factors in addressing this challenge is achieving an extended retention time and an optimal drug concentration at the desired site of action within the eye. In recent years, significant attention has been directed towards the development of polysaccharide-based in situ gelling systems as an attractive approach to enhancing the duration for which medications remain on the ocular surface. Ophthalmic in situ gelling relies on an aqueous solution containing stimuli-responsive polymers. Rapid in situ gelation occurs once these solutions are instilled into the eye in response to environmental changes, including pH, temperature, and ionic strength [114]. As a result, this approach offers several advantages, including enhanced adhesion to the ocular surface, extended retention time, sustained drug release, improved bioavailability, reduced systemic absorption, and the potential for less frequent dosing regimens.

Polysaccharides can be employed either individually or in combination with other responsive polymers to formulate ophthalmic in situ gels. An example is gellan, an ion-sensitive polymer, and its derivates, which undergo in situ gelation in response to mono- or divalent cations in tear fluid (e.g., Na^+^, Mg^2+^, and Ca^2+^). These formulations have been approved for the market in ophthalmic clinical application [115,116]. Similarly, alginate can form a gelled network in the presence of Ca^2+^ ions in the ocular surface [117]. Al-Juboori et al. developed a temperature-dependent in situ gelling system based on sodium alginate, incorporating ciprofloxacin and naproxen, which showed an enhanced retention time on the ocular surface [118]. Cellulose derivatives exhibit temperature-sensitive in situ gelling properties, with a phase transition temperature at around 35 °C, which aligns with the precorneal temperature [119,120]. Upadhayay et al. designed a chitosan-based nano in situ gel loaded with norfloxacin, offering the dual advantages of nanoparticles and an in situ gelling system for ocular drug delivery. This was achieved by using sodium tripolyphosphate (TPP) as a crosslinker and Carbopol 934 as the pH-sensitive trigger [121]. Furthermore, the incorporation of in situ gel formulations with colloidal carrier systems, such as lipid-based nanocarriers, has proven to be an effective strategy for increasing the bioavailability of ophthalmic drugs. Liu et al. developed an ocular nanogel loaded with curcumin (CUR) using chitosan-derived cationic nanostructured lipid carriers (CNLCs) and a thermosensitive gelling agent [122]. The results of in vitro release and corneal permeation indicated that the area under the curve (AUC) of apparent permeability was found to be 9.24-times higher than that of curcumin solution [122]. This significant improvement in bioavailability can be primarily attributed to increased viscosity and retention time on the corneal surface.

**Table 3:** Summary of recent studies on polysaccharide-based nanogels for overcoming ocular barrier.

Author	Type of Nanogel	Function and Mechanism	Size/Zeta Potential	Loaded Cargo	Type of Study	Year	Ref.
Buosi et al.	Chitosan	ARPE-19 cell targeting	~140 nm, 32 ± 2 mV	Resveratrol	In vitro	2020	[107]
Laradji et al.	Hyaluronic acid	Redox-responsive release, penetration	~80 nm, −7.56~−2.24 mV	Fluorescein	In vitro In vivo	2021	[108]
Zheng et al.	Catechol modified quaternized chitosan	Thermosensitive, tissue adhesive	/	/	In vitro In vivo	2020	[110]
Chaharband et al.	Chitosan, hyaluronic acid	Overcoming vitreous and retina barriers	/	siRNA	In vitro	2020	[112]
Silva et al.	Chitosan, hyaluronic acid	Enhanced muco-adhesion and permeation	≤300 nm, +30 mV	Erythropoietin	In vitro Ex vivo	2020	[113]
Lavikainen et al.	Gellan	Enhanced muco-adhesion and permeation	/	/	In vitro	2021	[116]
Nagai et al.	Methylcellulose	Prolonged pre-corneal and pre-conjunctival contact time	~93 nm	Tranilast	In vitro In vivo	2020	[119]

## 6. Polysaccharide-Based NGs to Overcome the Blood-Brain Barrier (BBB)

### 6.1. Characteristic of BBB

**Figure 4:**
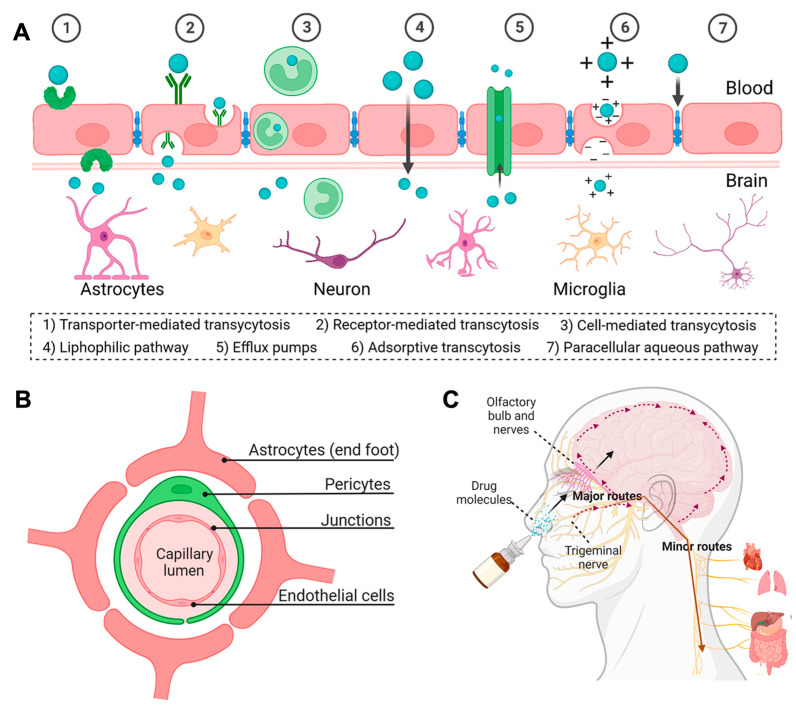
(**A**) Schematic diagram of different mechanisms for crossing the BBB. (**B**) Schematic diagram of the BBB structure. (**C**) Schematic illustration of the nose-to-brain administration route. Reprinted with permission from Wu et al. [125]. Copyright 2023 Signal Transduction and Targeted Therapy.

The blood-brain barrier (BBB) is a physiological protective barrier consisting mainly of a monolayer of brain endothelial cells in contact with vascular cells, glial cells, and neurons (Figure 4) [123]. Tight junctions between endothelial cells prevent potentially neurotoxic components and pathogens from the circulating blood from entering the central nervous system (CNS). However, while serving as a vital defense mechanism for the brain, the BBB also acts as a formidable barrier that excludes approximately 98% of small molecule drugs and nearly all macromolecular therapeutics from accessing the brain [124]. This presents a significant challenge for drug delivery to the central nervous system (CNS). Spurred by recent advancements in biomedicine and nanotechnology, various strategies for overcoming the BBB mainly involve the regulation of BBB permeability and the utilization of a brain-targeted drug delivery system. There are several pathways available for drug molecules to cross the BBB, including paracellular and transcellular diffusion, as well as transcytosis mediated by receptors, cells, transporters, or adsorption (Figure 4 A) [125]. Nevertheless, penetrating the BBB safely and effectively to achieve targeted drug delivery for CNS disorders, such as brain tumors, cerebrovascular diseases, and neurodegenerative diseases, remains a significant challenge.

### 6.2. Strategies for Overcoming the BBB

To overcome the BBB, an increasing number of advanced materials and technologies have emerged for enhanced brain-targeted drug delivery. Diverse nanocarrier-based drug delivery systems have been engineered to regulate and facilitate the crossing of the BBB for brain-targeted delivery of therapeutic agents, in a controlled and non-invasive manner, by using passive and/or active strategies. Polysaccharide-based carriers have shown enormous potential for BBB penetration, primarily owing to their biocompatibility, biodegradability, and muco-adhesion properties. Furthermore, several of these carriers have exhibited specific biological activities, including neuroprotection and therapeutic efficacy, making them valuable candidates for addressing various cerebral pathological conditions [126]. The presence of functional groups (e.g., hydroxyl, carboxyl, amino) in polysaccharides allows tailored chemical derivatization to enable BBB penetration and brain-specific targeting. For instance, nanocarriers based on chitosan and its derivates have been extensively explored for brain targeting due to several advantages, including their muco-adhesive properties resulting from electrostatic interactions, enhanced permeability through the modulation of tight junctions, and enhancement of the properties of other materials used in drug delivery systems [127,128,129]. Lipophilic molecules have a greater tendency to penetrate the BBB compared to hydrophilic molecules. Vashist et al. synthesized hydrophobically modified chitosan-hydroxyethyl cellulose by using a water-in-oil emulsion polymerization technique, which facilitated the nanogels in crossing an in vitro BBB model [130]. Remarkably, these biopolymeric nanogel particles exhibited auto-fluorescence at a wide range of wavelengths, suggesting their potential use as image-guided diagnostic and therapeutic tools for CNS applications [130].

Similarly, Azedi et al. developed methotrexate (MTX)-loaded nanogels using the ionic gelation of chitosan with polyanionic TPP. To enhance drug delivery to the brain, the surface of these MTX-loaded nanogels was modified with polysorbate 80 [131]. When administered intravenously in rats, both surface-modified and unmodified nanogels achieved significantly higher concentrations of MTX in the brain, in some cases exceeding tenfold, when compared to the free drug solution [131]. Pourtalebi et al. investigated the targeting delivery mechanism of these MTX-loaded chitosan-based nanogels to the BBB by utilizing a P-glycoprotein inhibitor. The results suggested that in the short term, the nanogels released the drug prior to the BBB, resulting in increased brain concentration. However, in the long term, the nanogels themselves may have the capability to cross the BBB [132]. Apart from cationic chitosan, Bostanudin et al. reported a series of anionic polysaccharides, including pullulan and guar, that have been modified with butylglyceryl groups. These modifications transform these polysaccharides into amphiphilic polymers capable of forming colloidal carriers through self-assembly [133]. In vitro studies demonstrated that butylglyceryl-modified pullulan enhanced drug transport across the BBB model membranes, making it a promising candidate for potential use in brain drug delivery [133].

In addition, intranasal administration is a non-invasive method for nose-to-brain drug targeting (Figure 4C). It enables the drug to bypass the BBB and be rapidly delivered directly to the brain through cellular and trans-neural pathways [125]. This approach offers several advantages for brain drug delivery, including rapid onset of action, minimal undesirable side effects, painless therapy, and non-invasiveness. However, intranasal drug delivery faces several challenges, including mucociliary clearance, limited permeation of hydrophilic compounds, and short retention time [134]. To address these limitations, polysaccharide-based formulations incorporating muco-adhesive agents and utilizing stimuli-responsive in situ gelling systems offer a promising strategy [134]. Gadhave et al. reported a strategy for glioma treatment by developing a nano lipid-based formulation loaded with teriflunomide (TFM) and incorporating gellan and carbopol. This innovative approach enhances the muco-adhesive capability and facilitates in situ gelling. The in vitro anticancer efficacy results were promising, with the in situ nanogel system exhibiting an IC_50_ of 7.0 μg/mL, nearly ten times lower than that of the TFM-loaded nano-lipid formulation (78.5 μg/mL). Furthermore, in vivo experiments showed promising results, demonstrating that the intranasal in situ nanogel formulation achieved drug concentrations in the brain that were two times higher than those achieved by intravenous administration of the nano lipid-based formulation. This enhanced permeability in intranasal delivery, attributed to the presence of gellan as a permeation enhancer, underlines the potential of this approach [135].

Moreover, Ourani-Pourdashti et al. prepared methotrexate (MTX)-loaded niosomal by using a modified reverse-phase evaporation method; this was then incorporated into a mixture of chitosan and Poloxamer 407 to produce a temperature-sensitive in situ gel formulation [136]. This chitosan-based in situ gel exhibited muco-adhesive properties, resulting in prolonged persistence in the nasal cavity and controlled drug release. In vivo study demonstrated higher drug concentrations in the brain and lower plasma concentration for the in situ gel formulation compared to other studied formulations, indicating its potential for effective brain drug delivery via intranasal administration. Table 4 summarizes the examples discussed in this section.

## 7. Conclusions and Future Perspective

The human organism possesses various biological barriers for preventing the entrance of foreign substances, including drugs for the treatment of diseases. These barriers pose a formidable challenge to achieving effective drug delivery. Polysaccharide-based nanogels have great significance in overcoming these barriers, thereby enhancing drug delivery and elevating bioavailability, due to their excellent biocompatibility and biodegradability, good modifiability, high drug encapsulation capacity, and specific biological activation. In this review, we first introduce the polysaccharide-based nanogels and outline recent advancements in drug delivery. Although polysaccharide-based nanogels have been developed for various biomedical applications, our focus here is primarily on their potential and recent progress in overcoming key biological barriers. These barriers encompass challenges related to mucus, the stratum corneum, ocular barriers, and the BBB. Polysaccharides or their derivatives, when used to create nanogels, can be employed individually or in combination with other polymers to achieve several objectives.

Nanogels based on polysaccharides or their derivates can be utilized alone or in incorporation with other polymers to enhance drug permeability, extend retention time, and/or specifically target the desired site, leading to overcoming biological barriers and effectively enhancing drug delivery. These benefits include improving drug permeability, prolonging retention time, and enabling precise targeting at specific sites, all of which collectively contribute to overcoming biological barriers and enhancing drug delivery effectiveness. Given the recent advancements in the field, polysaccharide-based stimuli-responsive in situ nanogels have attracted much attention due to their unique properties, which include enhanced adhesion and environment-triggered drug-release capabilities (e.g., pH, temperature, ionic, and redox conditions). These nanogels are finding promising applications, especially in ocular, dermal, and intranasal brain drug delivery. The intelligent responses exhibited by these nanogels represent a significant advancement when compared to conventional carrier systems. As a result, they are opening new avenues for the treatment of diseases, and particularly in overcoming challenging biological barriers.

However, it is worth noting that overcoming barriers and reaching the disease site is not the final step in the drug delivery process; on the contrary, it marks just another beginning of the journey [137]. Nanogels need to be internalized effectively to exert a therapeutic effect after administration. Recently, an increasing number of studies have been dedicated to exploring the design principles of nanocarriers that can enhance cellular uptake by modulating the mechanical property of nanocarriers, which represents a future exploratory research area [138,139,140]. Moreover, the current research primarily emphasizes the fascinating phase of exploring the experimental potential of nanogel-based polysaccharide carriers. One future challenge will be overcoming the translational gap between academic research and industrial development, which would ultimately deliver substantial benefits to patients.

## Figures and Tables

**Table 1:** Summary of recent studies on polysaccharide-based nanogels for overcoming mucus gel barrier.

Author	Type of Nanogel	Function and Mechanism	Size/Zeta Potential	Loaded Cargo	Year	Ref.
Mahmood et al.	Thiolated chitosan	Mucosal adhesion	26.30 ± 26.86 nm 0.03 mV	Curcumin	2017	[53]
Jin et al.	Thiolated chitosan	Mucosal adhesion	196.72 ± 0.45 nm 17.12 ± 0.50 mV	Bovine serum albumin	2022	[54]
Guaresti et al.	Thiolated chitosan	Mucosal adhesion	5–12 nm 36 ± 4 to −7 ± 1 mV	/	2020	[55]
Shtenberg et al.	PEG modified alginate	Mucosal adhesion	/	Ibuprofen sodium	2017	[57]
Tian et al.	Thiolated hyaluronic acid	Mucus penetration	Average 100 nm −26.2 ± 1.0 mV	INS	2018	[61]
Cao et al.	Chitosan	Mucus penetrationMuco-adhesive	/	Oral Vaccination	2021	[63]
Ways et al.	Chitosan	Mucus penetrating	145 ± 21 nm 15.0 ± 0.3 mV	/	2022	[65]

**Table 4:** Summary of recent studies on polysaccharide-based nanogels for overcoming BBB.

Author	Type of Nanogel	Function and Mechanism	Size/Zeta Potential	Loaded Cargo	Type of Study	Year	Ref.
Vashist et al.	Chitosan, hydroxyethyl cellulose	Auto-fluorescence, promote penetration	/	Bioactives	In vitro	2020	[130]
Pourtalebi Jahromi et al.	Chitosan	Enhanced pernetration	<200 nm, 22.8 ± 6.55 mV	Methotrexate	In vitro	2019	[132]
Bostanudin et al.	Guar, pullulan, chitosan	Enhanced permeation	120–200 nm, −23~−32 mV	Doxorubicin	In vitro	2020	[133]
Gadhave et al.	Gellan, Carbopol	Enhanced permeation, in situ gel, nose-to-brain delivery	117.80 nm, −21.86 mV	Teriflunomide	In vitro	2021	[135]
Ourani-Pourdashti et al.	Chitosan, Poloxamer 407	Temperature-sensitive in situ gel, nose-to-brain delivery	130.5 nm, −38.5 mV	Methotrexate	In vitro In vivo	2022	[136]

## Data Availability

Not applicable.
